# The Dynamic Properties at Elevated Temperature of the Thermoplastic Polystyrene Matrix Modified with Nano-Alumina Powder and Thermoplastic Elastomer

**DOI:** 10.3390/polym14163319

**Published:** 2022-08-15

**Authors:** Chih-Ming Chen, Huey-Ling Chang, Chun-Ying Lee

**Affiliations:** 1 Department of Mechanical Engineering, National Chin-Yi University of Technology, Taichung 41170, Taiwan; 2 Department of Chemical and Materials Engineering, National Chin-Yi University of Technology, Taichung 41170, Taiwan; 3 Graduate Institute of Manufacturing Technology, National Taipei University of Technology, Taipei 10608, Taiwan

**Keywords:** polystyrene, electronic packaging material, nano-alumina powder, thermoplastic elastomer, polymer based nanocomposites

## Abstract

The performance improvement of advanced electronic packaging material is an important topic to meet the stringent demands of modern semiconductor devices. This paper studies the incorporation of nano-alumina powder and thermoplastic elastomer (TPE) into thermoplastic polystyrene matrix to tune the thermal and mechanical properties after injection molding process. In the sample preparation, acetone was employed as a solvent to avoid the powder escape into surrounding during the mechanical mixing in a twin-screw mixer. The pressure and shear force were able to mix the composite with good uniformity in compositions. The samples with different compositions were fabricated using injection molding. The measured results showed that adding 5 wt.% of TPE into the simple polystyrene was able to raise the melt flow index from 12.3 to 13.4 g/10 min while the thermal decomposition temperature remained nearly unchanged. Moreover, the addition of small amount of nano-alumina powder could quickly improve the mechanical property by raising its storage modulus. For example, the addition of 3 wt.% of nano-alumina powder had an increase of 7.3% in storage modulus. Over doping of nano-alumina powder in the composite, such as 10 wt.%, on the other hand, lowered the storage modulus from 2404 MPa to 2069 MPa. The experimental study demonstrated that the tuning in the polystyrene’s thermal and mechanical properties is feasible by composition modification with nano-alumina powder and TPE. The better concentration of the additives should be determined according to the specific applications.

## 1. Introduction

Due to the stringent demands in computer and telecommunication devices today, such as speed performance, versatility in application, compact dimensions, etc., the packaging process for the semiconductor components has evolved from conventional techniques to the development of high power rate, high module density, compact size, and high precision process. The electronic packaging needs to provide the functions of configuration security and protection of the semiconductor devices in handling and service. Its life and reliability are greatly determined by the material used in the packaging process. In addition, the processability, heat dissipating property, and cost economy are also the important consideration factors for the packaging material. For packaging of electronic devices, the most common materials used are polymers. Because of the limited processing time for thermosetting polymers, thermoplastic resins are the common choices facilitated by their wide working temperature and easy fabrication. Moreover, the convenience in modifying the compositions of the material in rolling mixing to tune the desired properties further increases their advantages with lower manufacturing cost for using injection molding process. In this regard, nanocomposites with the capabilities in improving their mechanical and temperature endurance, electrical insulation, flame retardation, and optical transparency by the introduction of different fillers have attracted the attentions both from industry and academic societies [1].

Among the potential thermoplastic polymers, polystyrene, with its characteristics in light weight, good manufacturability, odorlessness, good coloration, electrical insulation, and chemical resistance, has been a popular option of industry. Its application has extended from conventional packaging in domestic compliances, transportation vehicles, mechanical products, and construction materials to high-tech 3C consumer electronics. In addition to its aforementioned characteristics, polystyrene is easily modified with fillers and reinforcements to meet the various applications. Since its first invention from the extraction from natural resin by the German Eduard Simon in 1839, polystyrene was commercially marketed until 1935 by the German BASF Corporation [2].

The molecular structure of polystyrene is composed of saturated carbon main chain with pendant conjugated phenyl rings. Its pendant group increases the irregularity of the molecular chain and reflects on its raised rigidity to mechanical loading. Subsequently, this high rigidity provides the material with high tensile strength and little apparent yielding with very low elongation, as compared with many of its polymeric counterparts. Therefore, its ejection molded parts are usually hard, brittle, and low in thermal resistance, which confine its wide use in industrial production. The low production cost of polymeric products is mostly derived from the easy manufacturability of ejection molding and thermal forming, etc. In injection molding, the fluidity of resin is one of the critical factors affecting the quality of the manufactured products. Since the abrupt change of resin’s fluidity in molding process can cause the clogging of injection nozzle, voids, and nonuniform heating, consequently, the manufactured part may suffer the insufficiency in mechanical strength [3].

In order to improve the mechanical performance and formability of the brittle polystyrene in injection molding, the composition modification using thermal elastomer filler had been attempted [4]. Elastomer is a class of viscoelastic polymers having low tensile modulus and high percent elongation. In its crosslinking molecular chain, the mechanical property can be adjusted by different combinations of the hard and soft segments. The hard segment enables the deformation recovery of the network structure after unloading. For the thermoplastic elastomer (TPE), the molecular structure contains both the rigid and flexible linkages, i.e., hard and soft linkages. The rigid segment provides the polymer with elasticity, mechanical performance at low temperature, hardness, tear strength, and modulus, etc., while the flexible one facilitates the emergence of crystallites formed by light chemical and physical crosslinking of hydrogen bonds between molecular chains. TPE possesses both the thermoplasticity and elasticity of polymer and reveals obvious thermogravimetric loss and viscosity variation at raised temperature [5].

Thermal elastomers usually provide better elasticity and easier fabrication through melting process than conventional crosslinked rubbers. Their structural characteristics propel the research and development in the material syntheses and potential applications. Dai et al. [6] directly synthesized polyethylene thermoplastic elastomers through α-diimine palladium catalyzed ethylene polymerization. Wang and coworkers [7] proposed the blending of B_4_C powder into polystyrene-block-poly(ethylene-ran-butylene)-block-polystyrene as the flexible shield. Kajita et al. [8] used triblock copolymer to synthesize hydrogen-bonded and ionically functionalized thermoplastic elastomers. Zhao and coworkers [9] studied the preparation of olefinic thermoplastic elastomer using peroxide-induced melt crosslinking.

As a different approach, polymeric composites with the incorporation of uniformly distributed nano-particles to transfer the loading in the matrix can provide the notable reinforcement in strength [10]. In appearance, alumina (Al_2_O_3_) is a white amorphous powder. Because of its high bonding strength between Al and O elements, alumina has the highest hardness and excellent dimensional stability among its oxide counterparts. Its good thermal conduction coefficient also facilitates the applications in devices which require good heat dissipation [6]. With its excellent heat resistance and high specific surface area, alumina powder is often employed as the carrier for high temperature applications [11,12,13,14]. However, the related works in the literature mostly concentrated on the development of new synthesis technologies in grafting and property improvement. The possible difficulties in product manufacturing and cost economy in industrial applications were not addressed extensively.

In contrast, the molecular structure of TPE constitutes of thermally reversible physical crosslinking, which resembles the molecular structure of rubber with chemical crosslinking. Therefore, TPE is recyclable and remoldable through melt extrusion and injection molding. Besides its rubber-like mechanical property, TPE also has good heat resistance, oil resistance, and wear resistance [15]. Moreover, the combination of super-elastic recovery capability from melt and liquid manifests the superior manufacturability and even the bearing strength for repetitive mechanical loading of TPE [16].

In this study, a thermoplastic polystyrene with intended application for electronic packaging is employed as the matrix material and further modification with additives of nano-alumina powder as reinforcement and TPE filler as toughener is systematically investigated. The composite material samples for the experimental testing were prepared through an injection molding process. The better composition of this polystyrene composite for advanced electronic packaging was explored. The thermoplastic nanocomposite proposed in this study not only could be prepared without resorting to chemical synthesis but also could be applied in the mass production of advanced electronic packaging process using thermoplastic ejection molding. The improvement in production quality and efficiency can be expected.

## 2. Experimental

### 2.1. Materials

A thermoplastic polystyrene with melt index (MI) of 12 g/10 min, heat deflection temperature (HDT) of 71 °C, and specific gravity of 1.05 was employed as the matrix material in this study. Two reinforcements—thermoplastic elastomer (TPE) and aluminum oxide (γ-Al_2_O_3_, alumina) nano-powder—were introduced into the composite. The former had an MI of 12 g/10 min and Shore A hardness of 88, while the latter had an average particle diameter of 50 nm and density 2.414 g/cm^3^.

### 2.2. Specimen Preparation

**Figure 1:**
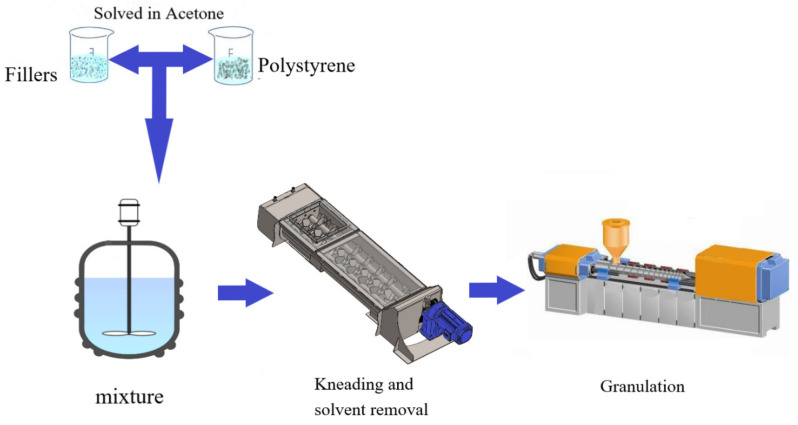
Schematic of preparation process.

Figure 1 presents the schematic diagram for the preparation of the specimens. The detailed process is described in the following:

(1) The weighted polystyrene granules and acetone solvent were sealed in a container for 24 h to dissolve the granules.
(2) The nano-alumina fillers and TPE were placed, respectively, inside a drying oven for 1 day to remove their water contents.
(3) The well dissolved polystyrene resin was mixed with the specified amount of nano-alumina filler in a twin-screw mixer for 10 min.
(4) With a heating rate of 2 °C/min, the temperature inside the chamber of the twin-screw mixer was raised gradually to 140 °C to remove the acetone solvent in the polystyrene resin. Subsequently, 5 wt.% of TPE was added into the mixing chamber, and agitation continued until a uniform mixture of composite was obtained.
(5) The well mixed polystyrene composite was removed from the mixer and cooled at room temperature for 1 day. The composite was then pulverized into granules.
(6) The prepared granules were fed into an injection molding machine to fabricate the specimens for characterization tests.
(7) Every specimen was rested at room temperature at least for 24 h before characterization test with each individual standard procedure.

### 2.3. Measurements

#### 2.3.1. Melt Flow Index

In the injection molding process of plastics, the resin is heated and transported in the screw extruder. The fluidity of the heated resin plays a key role in determining the quality of the molding process. This fluidity of the resin is usually determined by the melt flow index (MI) according to the test standard of ASTM D-1238 [17]. In this study, the temperature of the cylinder for the melt indexer was set at 200 °C, and the specimen was preheated for 5 min before the measurement. A 5 kg weight was applied on the piston, and the weight of the specimen passed the standard orifice (2.095 mm in diameter) during the 10 min period and was recorded.

A melt indexer is an instrument to measure the flow rate of polymer under loading. Higher MI index denotes the polymer has better fluidity or higher low rate. Because of its simplicity in measurement cost effective in instrument investment, melt indexer has been widely used by the industry in quality control or process monitoring of polymers. Moreover, it has also been employed to study the effect of dwelling time on the fluidity of polymers at processing temperature. It is understood that polymer can degrade in property when its exposure to high temperature prolongs. Therefore, a melt indexer can be applied in the determination of critical exposure duration of polymer to high temperature environment. Furthermore, the effect of adding lubricant and/or reinforcement in the polymer modification on the fluidity can also be the other applications. Generally, the measurement can be conducted through manual operation (Method A) or automation operation (Method B). In this study, Method B was adopted, and the following formula for calculating MI was used accordingly:

Melt Flow Index = (πγ^2^ L)/T × 600D (1)

In the above equation, γ, L, T, and D denote, respectively, the piston radius (cm), piston length (cm), testing duration (sec), and melt mass density (g/cc).

#### 2.3.2. Thermal Gravimetric Analysis

A thermogravimetric analyzer (TA-Q300 thermal analyzer, TA New Castle, DE, USA) was employed to measure the minute mass change of the heated polymeric specimen. A sample with mass of 7~10 mg was placed in a Pt dish enclosed in a nitrogen environment. The temperature of the chamber was raised from 25 to 800 °C in a heating rate of 10 °C/min, and the mass change during the heating process was measured by an in situ high precision balance. The associated chemical reaction or decomposition of the sample changed its mass. Accordingly, the thermal stability and the decomposition temperature of the sample can be measured.

#### 2.3.3. Dynamic Mechanical Analysis

The response of the specimen at different temperatures under swept sinusoidal mechanical excitation at different frequencies was performed by using a dynamic mechanical analyzer (DMA 2980, TA Instruments, New Castle, DE (USA)). A double-cantilevered specimen was subjected to a vibratory stress amplitude of σ and the corresponding steady state strain amplitude ε and phase δ were recorded as the frequency sweep. Accordingly, the following mechanical properties were calculated: [18]

Storage Modulus, E′= (σ/ε) cosδ(2)

Loss Modulus, E″= (σ/ε) sinδ(3)

Coefficient of Loss Tangent, Tan δ = E″/E′ (4)

(5) $$\mathrm{Complex}\;\mathrm{Modulus}{,}^{\;}E=\sqrt{{\left(\;E^{\prime }\right)}^{2}+({\left(\;E^{\prime \prime }\right)}^{2}}$$

Dynamic mechanical analysis (DMA) was performed according to ASTM D4065-01 to determine storage modulus (E′), loss modulus (E″), loss tangent (tan δ), and glass transition temperature (T_g_) of the composites. The tests were conducted in the dual cantilever beam mode with a vibration frequency of 1 Hz and a displacement amplitude of 10 µm in the DMA analyzer. The temperature was ramped from 30 to 150 °C at a rate of 2 °C/min. At least three specimens of each type were tested, and the data were analyzed. The DMA testing purpose was to simulate the loading conditions of the nanocomposite used in the electronic packaging and subjected to periodic vibration from the surroundings.

#### 2.3.4. Differential Scanning Calorimetry (DSC)

The measurement was conducted on a DSC machine (TA Instrument 2010) with 5~10 mg of sample sealed in aluminum pans under nitrogen atmosphere. A standard heating–cooling–heating cycle was used to evaluate crystallization and melting behavior of samples in a temperature range between 0 °C and 150 °C with a heating/cooling rate of 10 °C/min.

## 3. Results and Discussion

In this study, composite specimens were prepared by adding nano-alumina powder and/or TPE as the reinforcement and/or toughener in the polystyrene matrix. Specimen coupons were then fabricated by injection molding, and their properties were measured subsequently.

### 3.1. Molding Fluidity

According to testing standard of ASTM D-1238, melt flow indices of three specimens for each composition sample were measured, and their average and coefficient of variance (C.O.V.) were reported. Table 1 lists the test results for the melt flow index. For the processing of polymeric material, the lowering of MI after fabrication usually denotes the occurrence of extensive damage due to fabrication process. Insufficient fluidity of polymeric material during processing can result in sticking or blocking in the molding process. Subsequently, defects such as shortshots, voids, flowing marks, and welding lines appear in the molded part. On the other hand, polymeric melt with over fluidity can cause insufficient mixing, nonuniform coloration and flash, etc. Fluidity/viscosity of the polymeric melt usually closely relates to the length of its molecular chains. Polymer with longer chains improves its toughness and weatherability but reduces its crystallinity.

For the Type I samples listed in Table 1, which contain six different concentrations of nano-alumina powder, i.e., 1 wt.%, 2 wt.%, 3 wt.%, 4 wt.%, 5 wt.%, and 10 wt.%, in neat polystyrene (NPS) matrix, it is seen that MI decreases, more or less linearly, with the increase in alumina concentration. The addition of nano-alumina powder obviously decreased the fluidity of the composite. It can be understood that the inorganic alumina powder does not reach its melting point at the processing temperature. The polystyrene melt has to carry the alumina particles to flow toward the orifice opening, which causes more flow resistance and reduces the fluidity. It is noted that the C.O.V. of the MI measurement remains less than 5%, which signifies good repeatability in the specimen preparations. Nevertheless, the C.O.V. of 4.94% at high alumina concentration of 10 wt.% in PSA10 sample, comparing to the 1.08% of its NPS counterpart, still reveals the high amount in the addition of alumina powder to the polystyrene matrix could deteriorate the processing quality. This result suggests the composite with high nano-powder filler concentration should accompany the processing with longer mixing stage or increasing heating duration.

Type II composite is the further modification of the Type I with the addition of 5 wt.% linear thermoplastic elastomer (TPE). It is seen in Table 1 that the simple addition of 5 wt.% TPE in the neat polystyrene increased the MI from 12.31 g/10 min to 13.47 g/10 min, i.e., a 9.4% increase in fluidity from NPS sample to NPST sample. Furthermore, the addition of 5 wt.% TPE to PSA3 and PSA5 caused the increase in MI from 11.43 g/10 min and 11.09 g/10 min to 13.22 g/10 min and 12.99 g/10 min, respectively. In other words, there were 16.0% and 17.1% increases from PAS3 and PSA5 samples to NPTA3 and NPTA5 samples, respectively. All these results denote the TPE can be employed in this polymeric composite to compensate its fluidity decrement due to the incorporation of reinforcement filler in the material preparation. In the meantime, the influences from longer mixing duration and temperature exposure in processing can be alienated.

In the microscopic point-of-view, this TPE has physical properties similar to those of vulcanized rubber at room temperature, i.e., the molecular chains are constrained by the crosslinking chains, which can also deform elastically. At high temperature, these chains become plastically deformable, which facilitates the molding fabrication. More specifically, the molecular chain of TPE consists of both a rigid segment and a flexible segment. The rigid segment controls the elasticity, low-temperature property, hardness, tearing strength, and modulus, etc., while the flexible segment forms mild chemical and physical crosslinking via hydrogen bond and crystallization. The crosslinking in TPE molecular chains is physically thermal reversible which renders TPE recyclable and moldable by fusion injection and injection molding. Moreover, TPE demonstrates the super-elastic deformability, which facilitates the use of various fabrication processes and promotes its endurability to cyclic mechanical stress.

### 3.2. Thermogravimetric Analysis

A thermogravimetric analyzer was employed in the measurement of thermal property. Usually, the mass of the specimen decreases due to the evaporation of constituents upon heating. When the mass loss of the specimen reaches 5 wt.%, the corresponding temperature is defined as the thermal degradation temperature or Td_5%_ in notation. Similarly, Td_10%_ denotes the temperature associated with 10 wt.% mass loss of the sample. The derivative of the mass loss with respect to temperature represents the sensitivity of sample decomposition in the heating process. The temperature where the sample has maximum sensitivity in decomposition is denoted as maximum decomposition temperature (T_p_). At the end of decomposition, the sample reduces to char yield.

**Figure 2:**
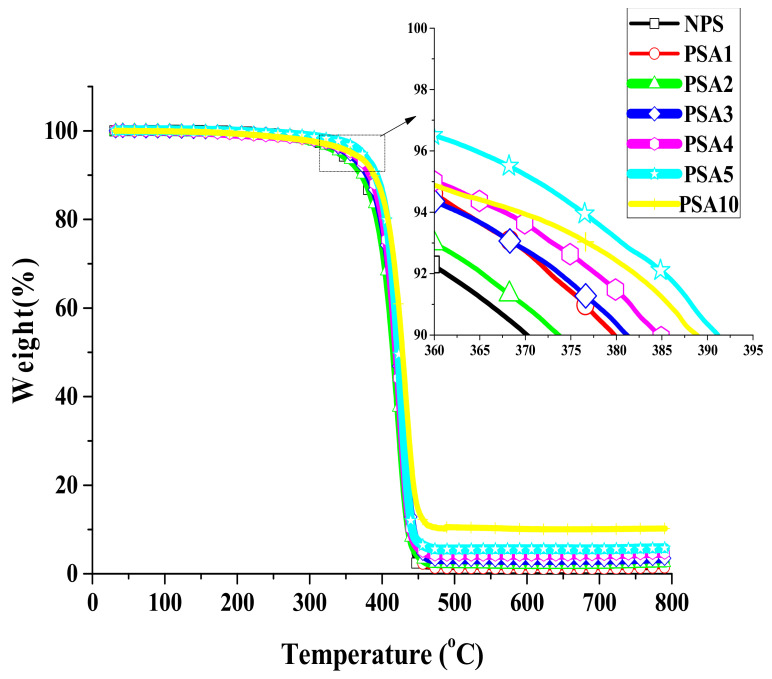
The relative weight change curves of Type I samples from TGA measurements.

**Figure 3:**
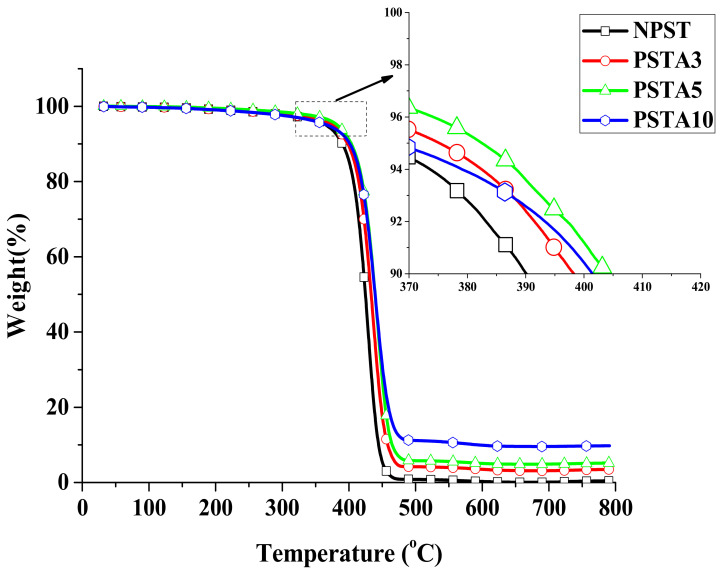
The relative weight change curves of Type II samples from TGA measurements.

Figure 2 and Figure 3 present the relative mass change curves in the TGA measurements for Type I and II samples, respectively. The important parameters from these measurements were distilled and are reported in Table 2. It is clearly seen that each curve in both figures has only one simple and smooth decreasing stage, which denotes no phase separation occurred in the measurement. Before the temperature reaching 340 °C, there was insignificant mass loss for both types of sample. The T_d5%_ for Type I samples with 1wt.%, 2 wt.%, 3 wt.%, 4 wt.%, 5 wt.%, and 10 wt.% of nano-alumina powder filler in polystyrene were 345.1, 356.6, 354.8, 360.1, 371.3, and 358.7 °C, respectively. Basically, composite samples with alumina filler showed higher thermal decomposition temperature than the pristine NPS sample. However, the increase in T_d5%_ did not correlate monotonously with the raise in concentration of alumina filler. The PSA10 sample only had similar T_d5%_ with its counterparts of PSA2. The result indicates adding small amount of alumina filler can have effective increase in the thermal resistance of the composite. Over filling of alumina in polystyrene matrix did not further raise its heat resistance as might be expected. As for the maximum decomposition rate, all samples showed their maximum decomposition temperatures fell within 424.1~434.1 °C, as listed in Table 2. The good thermal conductivity of alumina renders the faster thermal diffusion into the interior of the specimen and subsequently the lower Tp. Furthermore, the very low char yield of 1.23% for the NPS sample after heating up to 800 °C denotes a nearly complete decomposition. Therefore, the char yields for the other Type I samples listed in Table 2 were simply from the contribution of inorganic alumina filler. During the heating in TGA measurement, the molecular chains of the polymer broke into smaller molecules and evaporated. The organic parts turned into char residues under anoxic state and remained as the yield with the inorganic alumina after the test. Therefore, the residual char increased with the alumina content in the composite sample.

For the Type II samples with the additional addition of 5 wt.% TPE in their corresponding Type I samples, their relative mass change curves are shown in Figure 3 and the important parameters are listed in Table 2. It was found that all thermal decomposition temperatures for Type II samples were raised from their Type I counterparts. Among them, the highest thermal decomposition temperature occurred for the PSTA5 sample. Its T_d5%_ had a raise of nearly 41 °C from the pristine polystyrene matrix (NPS). The incorporation of TPE in the composite did not diminish its thermal resistance.

### 3.3. Dynamic Viscoelasticity Analysis

**Figure 4:**
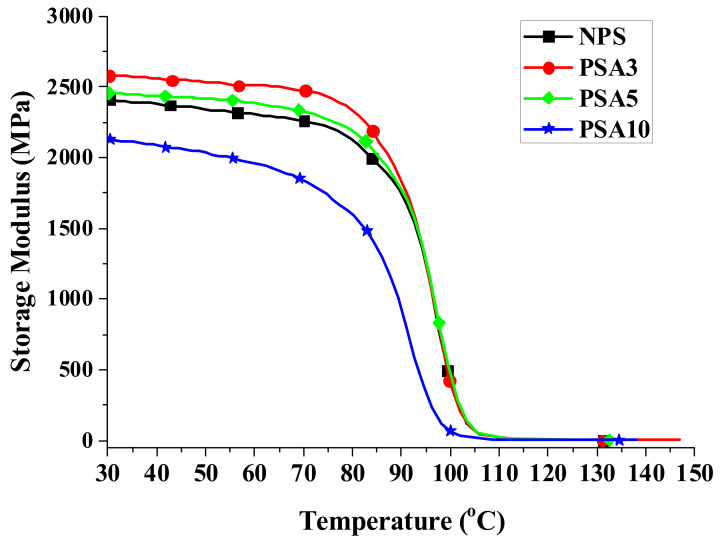
The measured storage moduli of Type I samples with respect to the temperature changes.

**Figure 5:**
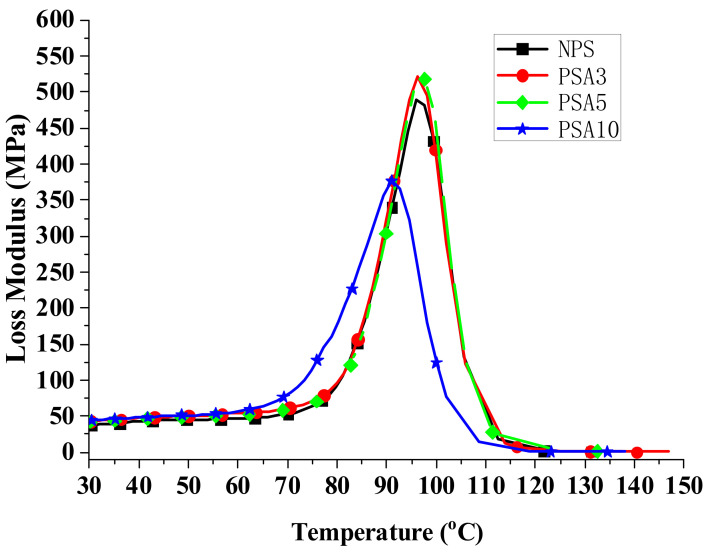
The measured loss moduli of Type I samples with respect to the temperature changes.

**Figure 6:**
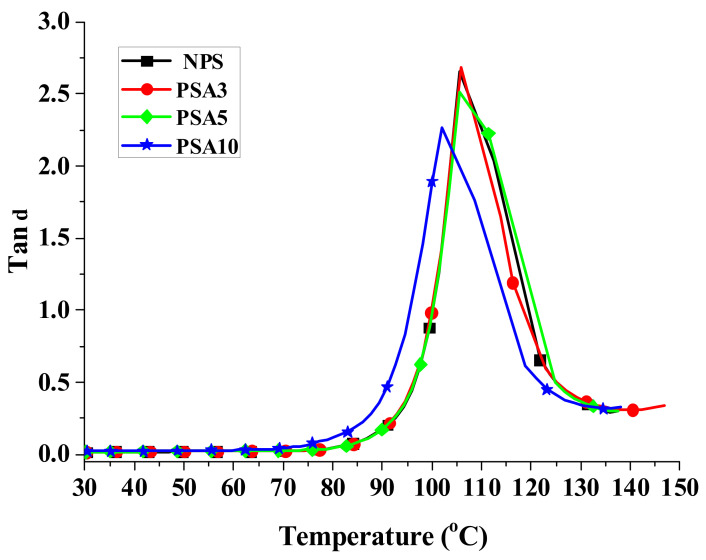
The measured loss tangent of Type I samples with respect to the temperature changes.

When a material is used in dynamic loading, its material response is usually characterized by viscoelastic properties, which are measured by using a DMA analyzer, as mentioned in Section 2.3.3. Figure 4, Figure 5 and Figure 6 present, respectively, the measured storage moduli (E′), loss moduli (E″), and loss tangents (Tan δ) of Type I samples with respect to the temperature changes. It is known that E′, E″, and Tan δ denote the elastic, viscous, and viscoelastic behaviors of the material, respectively. The temperature at which the Tan δ attains maximum is defined as the glass transition temperature (T_g_). In other words, when heated across T_g_ the material changes from glassy state to rubber-like state. At T_g_, storage modulus E′ has a sharp drop and material damping reaches maximum.

As seen in Figure 4, the storage modulus slightly decreases at low temperature in heating for each sample. Adding small amount of nano-alumina powder to the pristine polystyrene matrix can effectively increase the storage modulus while this increase in storage modulus diminishes for the over filling sample (PSA10). This change of storage modulus from 2404 MPa of NPS to 2580 MPa of PSA3 and 2069 MPa of PSA10 is listed in Table 3. Adding 3 wt.% of nano-alumina powder into polystyrene had an increase of 7.3% in storage modulus. The drop of storage modulus for the PSA10 sample could be caused by the incomplete bonding between the nano-powders and the matrix due to the high filler content.

**Figure 7:**
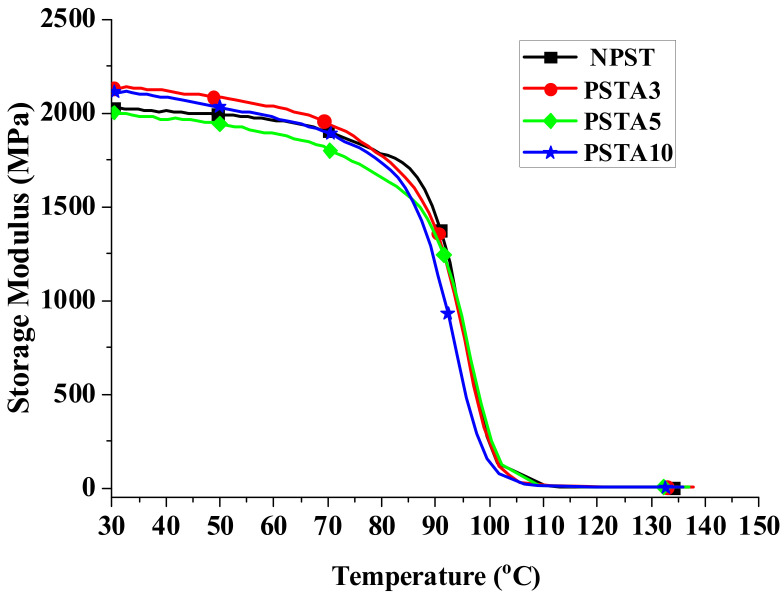
The measured storage moduli of Type II samples with respect to the temperature changes.

**Figure 8:**
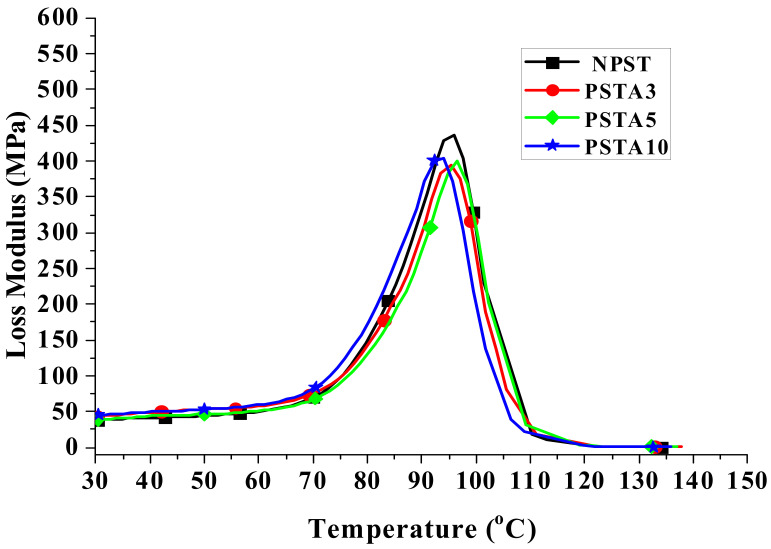
The measured loss moduli of Type II samples with respect to the temperature changes.

**Figure 9:**
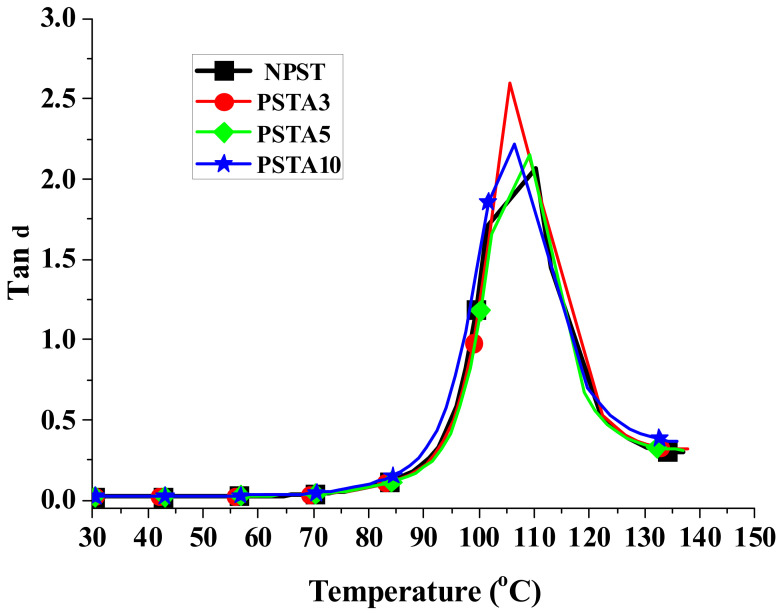
The measured loss tangent of Type II samples with respect to the temperature changes.

The loss modulus presented in Figure 5 represents the material’s viscous characteristic and energy dissipation capability. The loss tangent shown in Figure 6 has similar trend with loss tangent. They all reach maximum at glass transition temperature. The glass transition temperatures of PSA3 and PSA5 samples remained nearly unchanged with their NPS counterpart while the one of PSA10 decreased noticeably. Moreover, the full width at half maximum (FWHM) of the Tan δ curve increased basically with the addition of alumina filler (PSA5 and PSA10) to the polystyrene matrix (NPS). This result denotes microscopically the motion of molecular chains of polystyrene was restrained by the alumina particles. As mentioned previously, the incomplete bonding of nano-alumina powder with the polystyrene matrix for the PSA10 sample might reduce the extent of viscous motion for the molecular chains and lower the peak magnitudes of both the loss modulus and loss tangent curves. These important material parameters are also summarized in Table 3. Similar measured results for the Type II samples are presented in Figure 7, Figure 8 and Figure 9.

**Figure 10:**
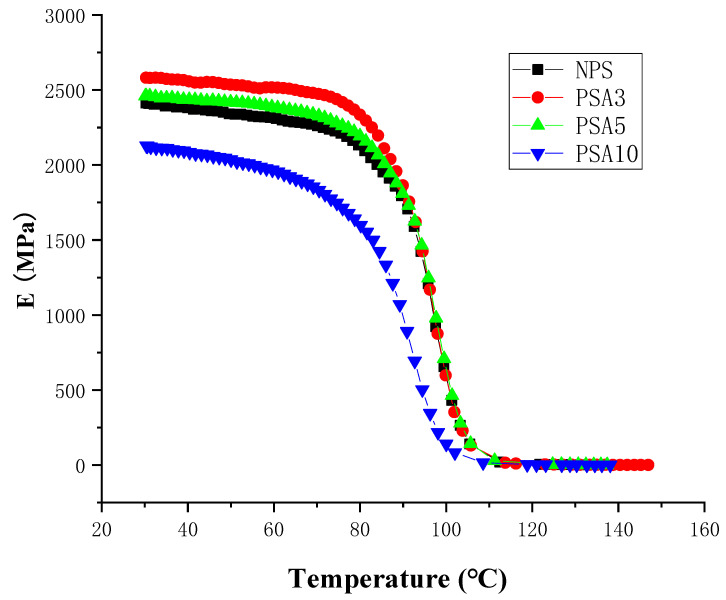
The measured complex moduli of Type I samples with respect to the temperature changes.

**Figure 11:**
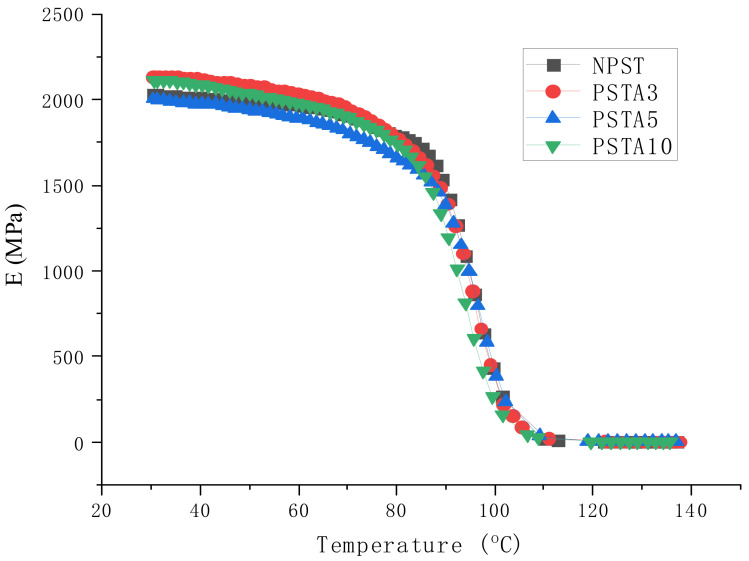
The measured complex moduli of Type II samples with respect to the temperature changes.

With the elastic and loss moduli obtained, the magnitudes of complex modulus of the samples can be calculated according to the formula of Equation (5). For the material under static loading, its Young’s modulus is usually determined from the linear slope of the stress-strain curve within elastic limit. On the contrary, under dynamic loading the capability of the material’s resistance to cyclic loading is represented by the measured complex modulus at different temperatures. The results of the composite samples in this study are shown in Figure 10 and Figure 11. At low temperature, the high magnitude of E indicated the material’s high rigidity under glassy state. As the temperature was raised, the motion of the molecular chain increased which manifested through the accompanying increase in viscous behavior. Part of the loading energy was consumed in overcoming the internal friction among the molecular chains. Near the glass transition temperature, this interactive reaction from the internal friction accelerated the decrease in complex modulus. This temperature dependent response of the viscoelastic material is important information for its engineering application.

### 3.4. Differential Scanning Calorimetry Measurements

**Figure 12:**
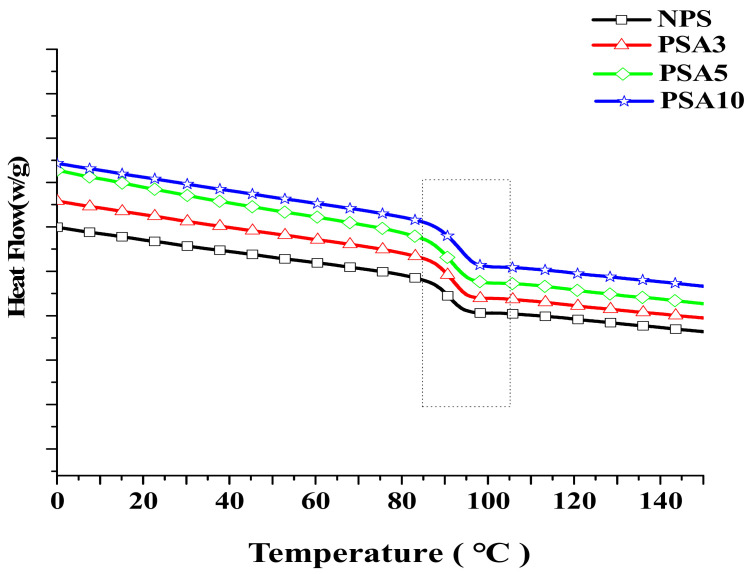
The measured heat flow curves from DSC for the Type I samples.

Figure 12 presents the measured heat flow curves from DSC for the Type I samples. The abrupt change in the slope of the heat flow curve denotes the occurrence of phase change. For this polymeric material system, it represents the phase change from glassy state to rubbery state while heated. More specifically, the molecular chains in the amorphous microstructure are more restrained in motion at low temperature and reflect to the glassy and brittle state in macroscopic behavior. Accompanying the raise in temperature, the heat flow curve changes in the endothermic direction. At a certain temperature range, the said molecular chains gain enough energy to move more easily and reflect to the rubbery behavior of the material macroscopically. This glass transition temperature Tg was recorded and is listed in Table 4.

The results in Figure 12 reveal that the heat flow increased with the raise in alumina content of the sample. The intrinsic characteristics of alumina in good thermal conductivity and electrical resistance facilitate its application in advanced electronic packaging. Nevertheless, the incorporation of inorganic particles interferes with the flexible movement of the molecular chains. Tg increases accordingly as seen from 96.6 °C of NPS sample to 98.4 °C of PAS10 sample. This result also demonstrates that adding alumina nano-powder is a feasible method to tune the composite’s thermal property.

**Figure 13:**
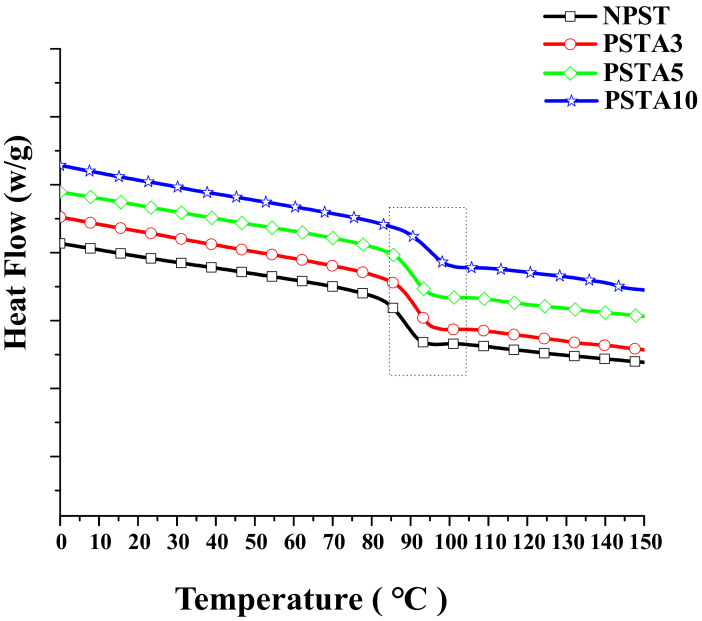
The measured heat flow curves from DSC for the Type II samples.

For Type II samples, the heat flow curves are shown in Figure 13 and their Tgs are also listed in Table 4. As it is seen in Table 4, the further addition of 5 wt.% of TPE lowered the Tgs of their counterparts. This should be caused by the less motion resistance of the molecular chains in the polymeric matrix owing to the soft and flexible TPE. The similar restraint in the movement of molecular chain due to the addition of alumina particles was prevailed in Type II samples as seen from the increasing Tg with alumina content. However, a small amount of alumina doping in the composite can find obvious effect. Other study also reported the doping of nanoparticles could impose more restraint on the movement of atoms in the metallic matrix and alleviate the friction and wear of the composite [19].

The above results show the addition of inorganic nano particles in the polymeric matrix can impose more restraint on the movement of molecular chains and raise the Tg of the composite. Moreover, adding thermoplastic elastomer can be helpful for facilitating the flexible motion of the molecular chains and providing the tunability on the complex modulus of the composite. The use of nano-alumina particles in this study, with its good thermal conductivity, electrical insulation property, and cost effectiveness, presents a potential candidate material in advanced electronic packaging applications.

### 3.5. Microscopic Morphology

**Figure 14:**
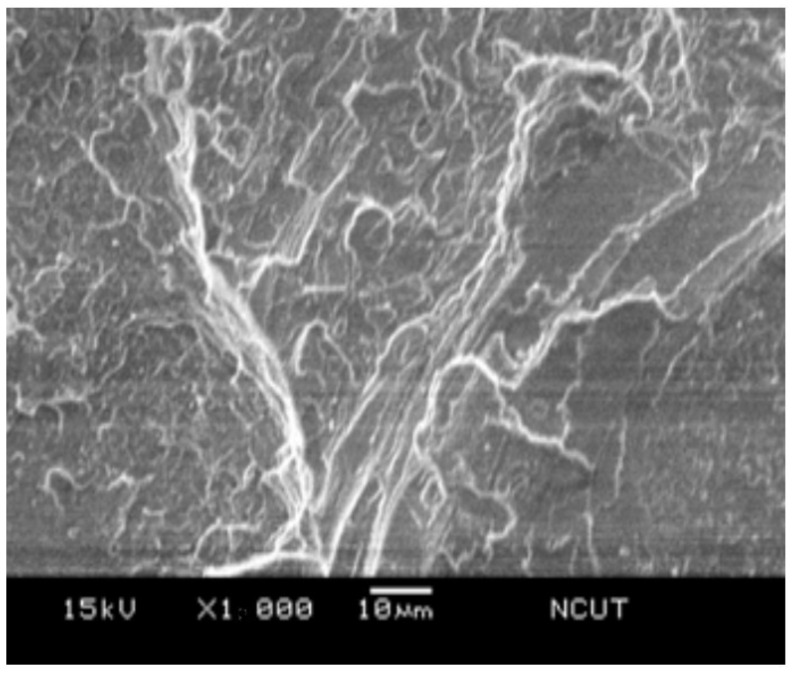
SEM micrograph of the fractured surface on NPS sample.

**Figure 15:**
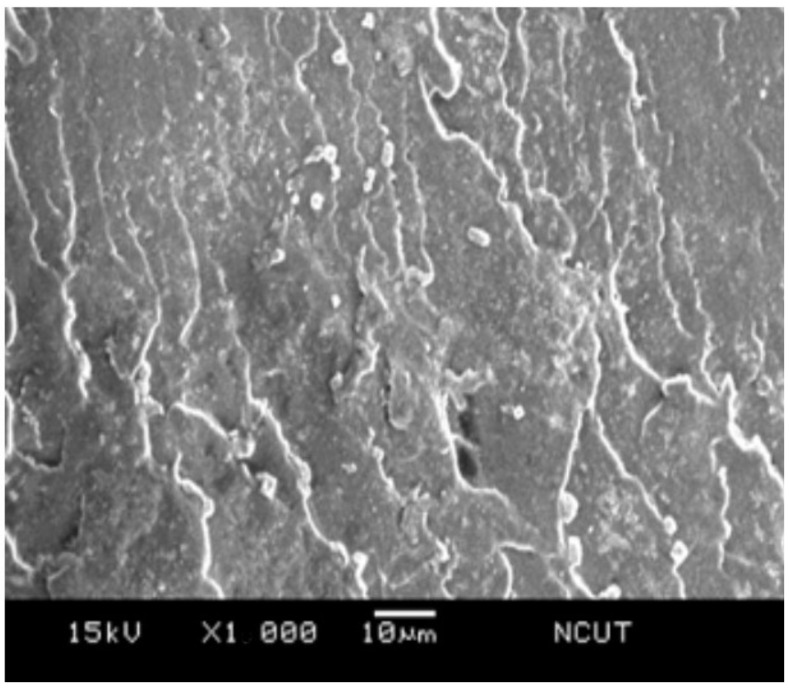
SEM micrograph of the fractured surface on PSA5 sample.

**Figure 16:**
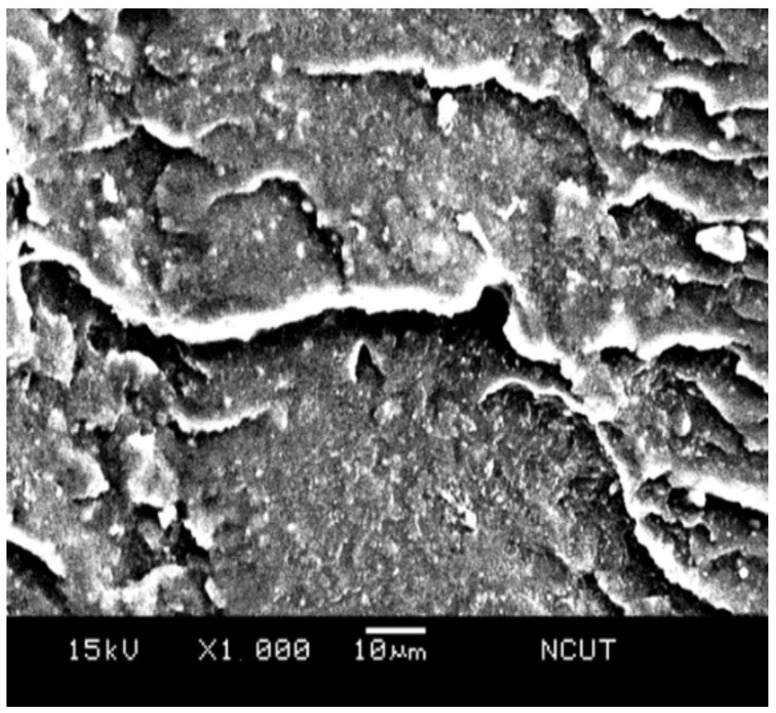
SEM micrograph of the fractured surface on PSA10 sample.

In order to examine the microscopic morphology in the prepared samples, the fractography of the sample after Izod impact testing was investigated using scanning electron microscope (SEM). Figure 14 presents the SEM micrograph of the NPS sample, i.e., the pristine matrix. The fractography revealed the radial tearing wrinkles, which confirmed the brittle nature of the polystyrene resin. For the PSA5 and PSA10 samples, which were the ones with the additions of 5 wt.% and 10 wt.% from their NPS counterparts, respectively, their fractographs are shown in Figure 15 and Figure 16. Both graphs demonstrated shorter tearing wrinkles, no radial emergence, and the appearance of particulate/void structure. We realized that the high impact resistance of the PSA samples resulted from the disruption in impact crack growth with the existence of alumina particles. Moreover, the weaker bonding between alumina particle and matrix also created the separation of the particle from the matrix and left the void on the fractured surface. The population of the void increased with the alumina content in the composite.

**Figure 17:**
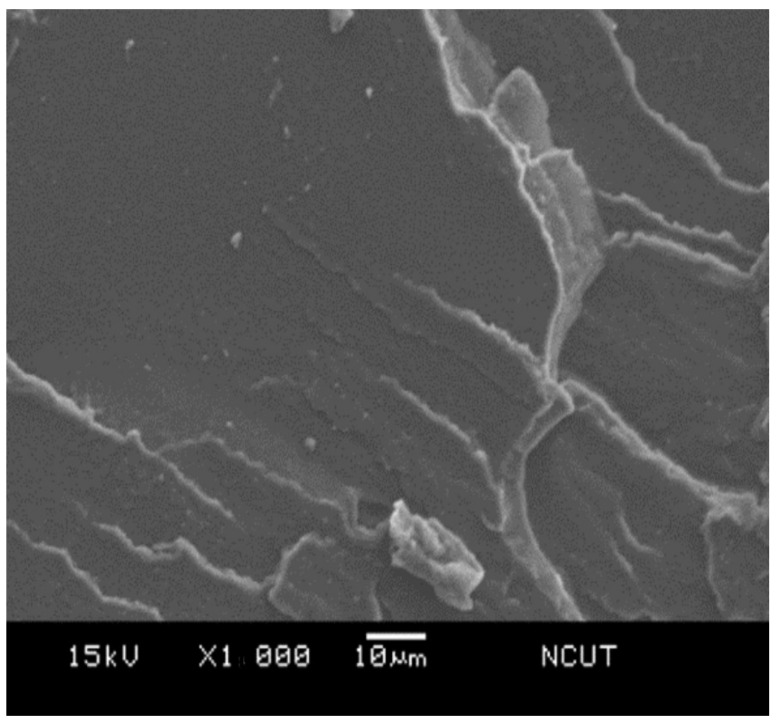
SEM micrograph of the fractured surface on NPST sample.

**Figure 18:**
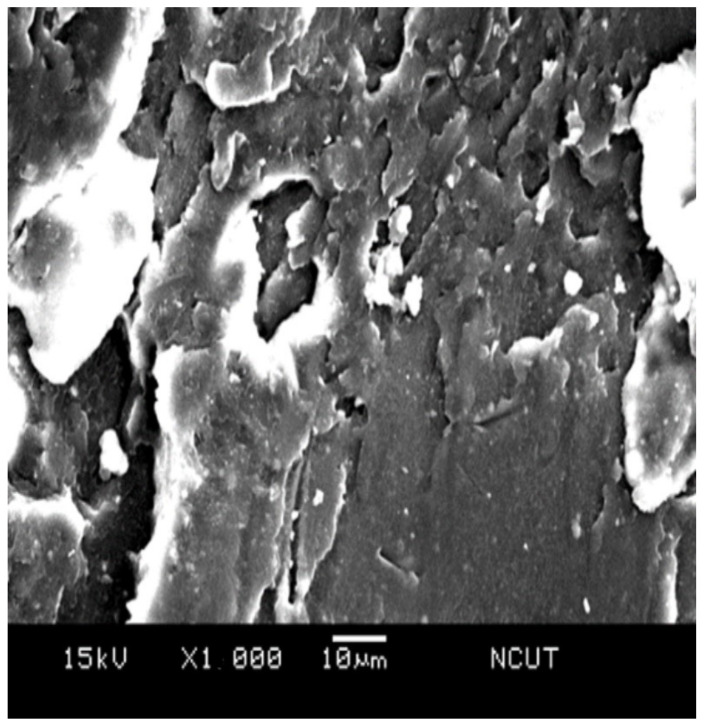
SEM micrograph of the fractured surface on PSTA3 sample.

With the simple addition of 5 wt.% TPE in the matrix, most of the radial tearing wrinkles disappeared, and a larger portion of flat area emerged on the surface, as seen in Figure 17. Both polystyrene and TPE mixed uniformly, and the hard and brittle property of the polystyrene changed. With further doping of 3 wt.% of alumina particles, i.e., PSTA3 sample, Figure 18 shows the fractograph having randomly oriented cracks, flake segments, and particulate/void structure. The rougher surface morphology denotes the sample can absorb more impact energy.

## 4. Conclusions

The effects of adding nano-alumina powder and thermoplastic elastomer into polystyrene to modify its thermal and mechanical properties were investigated. Usually, the conventional mixing of nano-powder into polystyrene matrix renders a nonuniform mixture with degraded thermal and mechanical properties. Alternatively, using twin-screw mixer in blending often causes the escape and pollution of the powders. The proposed method of dissolving the polystyrene pallets first with acetone and subsequently mixing them with filler powder in the screw mixer overcame the difficulty and obtained a uniformly composite material. The obtained composite, with the resin to transfer the loading stress among the filler particles and the inorganic particles serving as the reinforcement, provided improved mechanical properties.

This composite material can be palletized and employed in the packaging of electronic devices through injection molding manufacturing process. As a potential candidate for packaging electronic devices, the thermal and mechanical properties would be critical. Therefore, the melt flow index, thermal stability, and dynamic properties in vibratory loading of this polystyrene composite should be improved.

From the measured MI index of the composite samples, the addition of 5 wt.% TPE was able to raise the resin fluidity by 9.42% from its pristine polystyrene counterpart. Moreover, the doping of 5 wt.% TPE into the composite having 3 wt.% of nano-alumina powder increased its fluidity by 16%. Both results demonstrated that 5 wt.% TPE additive in the either pristine polystyrene or nano-alumina/polystyrene composite improved the melt fluidity and facilitated the manufacturability in injection molding process. As for thermal stability observed from TGA measurements, it was found that adding 5 wt.% of nano-alumina powder was capable of obtaining the highest degrading temperature for the composite. Over doping, such as 10 wt.% of nano-alumina powder, lowered the degrading temperature. It was noted from the comparison between Type I and Type II samples that adding 5 wt.% of TPE did not influence the thermal degrading temperature notably. In the dynamic mechanical properties measured, the results revealed that the addition of small amount nano-alumina powder to the polystyrene could increase the storage modulus and the mechanical property. More specifically, adding 3 wt.% of nano-alumina powder was able to obtain a maximum storage modulus at 2580 MPa, which was a 7.3% increment from its polystyrene matrix counterpart. On the other hand, the doping of TPE in the composite revealed the slight decrease in the complex moduli or reduction in the brittleness of the polystyrene. The results from this study demonstrate the property modification on the polystyrene resin by tailoring the compositions with either nano-powder or thermoplastic elastomer filler is a feasible approach. Of course, the suitable amount of doping should be determined by the individual application.

## Figures and Tables

**Table 1:** The measured melt flow indices of the composite samples prepared with different compositions.

Sample Name		Compositions			Melt Index (g/10 min)				
		PS (wt.%)	Al_2_O_3_ (wt.%)	TPE (wt.%)	No.	No.	No.	Avg.	C.O.V.
					(a)	(b)	(c)		(%)
Type I	NPS	100	0	0	12.12	12.32	12.50	12.31	1.54
	PSA1	99	1	0	11.98	12.05	12.50	12.18	2.32
	PSA2	98	2	0	11.84	12.00	11.82	11.89	0.83
	PSA3	97	3	0	11.55	11.54	11.20	11.43	1.74
	PSA4	96	4	0	11.50	11.32	11.00	11.27	2.25
	PSA5	95	5	0	11.00	11.03	11.23	11.09	1.13
	PSA10	90	10	0	10.50	9.92	9.52	9.98	4.94
Type II	NPST	95	0	5	13.40	13.76	13.24	13.47	1.98
	PSTA3	92	3	5	13.22	12.94	13.50	13.22	2.12
	PSTA5	90	5	5	12.82	13.00	13.15	12.99	1.27
	PSTA10	85	10	5	11.85	12.10	11.92	11.96	1.08

**Table 2:** The important parameters obtained from TGA measurements of samples prepared in this study.

Sample Name		Compositions			Thermal Decomposition Temperature		Maximum Decomposition Temperature	Char Yield
		PS (wt.%)	Al_2_O_3_ (wt.%)	TPE (wt.%)	T_d5%_ (°C)	T_d10%_ (°C)	T_p_ (°C)	(wt.%)
Type I	NPS	100	0	0	341.4	370.2	434.1	1.23
	PSA1	99	1	0	345.1	374.9	425.6	1.46
	PSA2	98	2	0	356.6	378.8	424.1	2.51
	PSA3	97	3	0	354.8	381.2	427.6	3.52
	PSA4	96	4	0	360.1	384.7	425.1	4.72
	PSA5	95	5	0	371.3	391.2	427.0	5.79
	PSA10	90	10	0	358.7	388.8	432.3	10.24
Type II	NPST	95	0	5	365.9	390.1	436.9	0.42
	PSTA3	92	3	5	375.2	398.2	439.2	3.46
	PSTA5	90	5	5	382.5	403.8	438.7	5.18
	PSTA10	85	10	5	367.7	401.5	442.1	9.80

**Table 3:** The measured viscoelastic properties of samples.

Sample Name		Storage Modulus at 32 °C (MPa)	Loss Modulus at 32 °C (MPa)	T_g_ (°C)	FWHM (°C)
Type I	NPS	2404	38.09	105.47	17.69
	PSA3	2580	43.68	105.75	14.42
	PSA5	2455	42.33	105.69	19.34
	PSA10	2069	45.32	102.05	19.84
Type II	NPST	2024	38.74	110.31	20.53
	PSTA3	2137	44.75	105.55	18.07
	PSTA5	1998	39.40	109.06	19.45
	PSTA10	1974	43.98	105.34	21.20

**Table 4:** The important parameters obtained from DSC measurements of samples prepared in this study.

Sample Name		Compositions			Glass Transition Temperature
		PS (wt.%)	Al_2_O_3_ (wt.%)	TPE (wt.%)	Tg (°C)
Type I	NPS	100	0	0	96.6
	PSA3	97	3	0	97.3
	PSA5	95	5	0	98.3
	PSA10	90	10	0	98.4
Type II	NPST	95	0	5	88.6
	PSTA3	92	3	5	92.0
	PSTA5	90	5	5	93.0
	PSTA10	85	10	5	96.5

## Data Availability

The data underlying this article will be shared on reasonable request from the corresponding author.
